# Simple Reversed-Phase HPLC Method with Spectrophotometric Detection for Measuring Acetaminophen-Protein Adducts in Rat Liver Samples

**DOI:** 10.1100/2012/145651

**Published:** 2012-04-19

**Authors:** Miteshkumar Acharya, Cesar A. Lau-Cam

**Affiliations:** Department of Pharmaceutical Sciences, College of Pharmacy and Allied Health Professions, St. John's University, Jamaica, NY 11439, USA

## Abstract

A simple reversed-phase HPLC method for measuring hepatic levels of acetaminophen- (APAP-) protein adduct following an overdose of APAP was developed. An aliquot of liver homogenate in phosphate-buffered saline pH 7.4 (PBS) was placed on a Nanosep centrifugal device, which was centrifuged to obtain a protein residue. This residue was incubated with a solution of *p*-aminobenzoic acid (PABA), the internal standard, and bacterial protease in PBS, transferred to a Nanosep centrifugal device, and centrifuged. A 100 **μ**L portion of the filtrate was analyzed on a YMC-Pack ODS-AMQ C18 column, using 100 mM potassium dihydrogen phosphate-methanol-acetic acid (100 : 0.6 : 0.1) as the mobile phase, a flow rate of 1 mL/min, and photometric detection at 254 nm. PABA and APAP-cystein-S-yl (APAP-Cys) eluted at *~*14.7 min and 22.7 min, respectively. Method linearity, based on on-column concentrations of APAP-Cys, was observed over the range 0.078–40 **μ**g. Recoveries of APAP-Cys from spiked blank liver homogenates ranged from *~*83% to 91%. Limits of detection and of quantification of APAP-Cys, based on column concentrations, were 0.06 **μ**g and 0.14 **μ**g, respectively. RSD values for interday and intraday analyses of a blank liver homogenate spiked with APAP-Cyst at three levels were, in all cases, ≤1.0% and <1.5%, respectively. The proposed method was found appropriate for comparing the antidotal properties of N-acetylcysteine and taurine in a rat model of APAP poisoning.

## 1. Introduction

In addition to being the most common analgesic and antipyretic agent worldwide, acetaminophen (N-acetyl-p-aminophenol, APAP) is also regarded as a leading cause for intentional or unintentional overdosing, for severe hepatotoxicity and for acute live failure (ALF) in the United States and the United Kingdom [1, 2]. One of the critical contributory steps to the hepatotoxicity that follows the ingestion of overdoses of APAP is its initial oxidative metabolism to N-acetyl-*p*-benzoquinoneimine (NAPQI) by hepatic cytochrome P450 isoforms and subsequent covalent binding of this reactive metabolite to hepatic cytosolic and organelle proteins, primarily, though, not exclusively, to cysteine residues to generate stable 3-(cystein-S-yl)acetaminophen (APAP-Cys) adducts [3]. The extent to which APAP binds to hepatic proteins is of clinical interest since it reflects the severity of its dose-related damaging action on the liver, especially to centrilobular cells [4] and is a way to improve diagnostic accuracy in patients with ALF [5]. Analytical methods presently available to measure APAP covalently bound to proteins in the liver and serum have included an avidin-biotin amplified competitive enzyme-linked immunosorbent (A-B ELISA) assay [6, 7], an immunoblot technique using antiserum specific for APAP-CYS and imaging densitometry [8, 9], and a high-performance liquid chromatography with coulometric electrochemical detection (HPLC-ECD) [5, 10]. While the A-B ELISA and immunoblot techniques require multiple procedural steps and reagents and an antiserum specific for APAP-Cys that is not readily available, the HPLC-ECD method relies on a lengthy dialysis step to separate protein-bound APAP from any interfering amino acids, unbound APAP, or free APAP-Cys and on an electrochemical detection setup that may not be available in the average laboratory. A more recently published liquid chromatographic method coupled with tandem mass spectrometric has only been evaluated as a qualitative tool for detecting the binding of APAP to serum albumin [11].

In comparison with currently available analytical methods for measuring APAP-protein conjugates in biological samples, the HPLC method with photometric detection described in the present report is methodologically simpler, requiring less procedural steps, and using readily available reagents. The sample preparation has been greatly simplified by utilizing centrifugal ultrafiltration to initially isolate APAP-protein adducts, and, subsequently, the APAP-Cys fragments liberated by enzymatic hydrolysis from their anchoring proteins. The method has been found to be specific, reproducible, and with the required sensitivity to detect the levels of APAP-Cys associated with an overdose of APAP. Using the rat as an experimental model, the proposed HPLC method was found to be not only useful for measuring APAP-protein adducts in the liver but also for assessing the antidotal effectiveness of model compounds against APAP-induced hepatotoxicity due to a toxic dose of APAP.

## 2. Experimental

### 2.1. Materials and Solvents

APAP, *p*-aminobenzoic acid (PABA), potassium dihydrogen phosphate, and protease (Pronase E, type XIV from *Streptomyces griseus*, 4 units/mg of solid) were purchased from Sigma-Aldrich, St. Louis, MO, USA. 3-Cysteinylacetaminophen (APAP-Cys) trifluoroacetic acid salt was purchased from Toronto Research Chemicals Inc., North York, ON, Canada. HPLC-grade methanol, HPLC-grade water, perchloric acid (70%, w/v), and glacial acetic acid were purchased from J. T. Baker, Phillipsburg, NJ, USA. Nanosep centrifugal device with Omega ultrafiltration membrane molecular weight cutoff 30 kDa, red type, was from Pall Corporation, East Hills, NY, USA; and high-grade regenerated cellulose tubular dialysis membrane (Cellu-Sep H1, nominal MWCO 3500) was from Membrane Filtration Products, Inc., Seguin, TX, USA.

### 2.2. HPLC System

Chromatographic analyses were carried out on a Waters Alliance HPLC system consisting of the 2695 Separations Module, 2998 photodiode array detector and 2707 autosampler (Waters Corporation, Milford, MA, USA). The chromatograms were acquired with an Empower Chromatography Software (Waters Corporation, Milford, MA, USA).

### 2.3. Chromatographic Conditions

The chromatographic separations were achieved on an YMC-Pack ODS-AMQ C18, 150 × 4.6 mm i.d., 5 *μ*m, column (Waters Corporation, Milford, MA, USA). The mobile phase was a mixture of 100 mM potassium dihydrogen phosphate-methanol-acetic acid (100 : 0.6 : 0.1, by volume), pH 4.7 ± 0.2, filtered in vacuo through a 0.45 *μ*m membrane filter (Millipore, Bedford, MA, USA), and degassed by sonication prior to use. The mobile phase was delivered at a rate of 1 mL/min. A 100 *μ*L volume of sample was injected from an autosampler kept at 4°C. The detection wavelength was 254 nm.

### 2.4. Solutions

#### 2.4.1. Protease Solution

This solution was prepared by dissolving a sample of lyophilized, powdered, bacterial protease in phosphate-buffered saline (PBS) pH 7.4 to obtain a solution containing 8 units per mL.

#### 2.4.2. PABA Solution

This solution was prepared by dissolving a sample of PABA, the internal standard, in distilled water to obtain a solution containing 1 mg/mL.

### 2.5. Validation of the Method

#### 2.5.1. Linearity and Recovery

The linearity of the proposed HPLC method was assessed by preparing two calibration curves, one in protein matrix and one in distilled water, from serial dilutions of an aqueous APAP-Cys stock solution (1 mg/mL). The protein matrix was obtained by placing 0.5 mL aliquots of a homogenate made from the liver of a rat treated only with 50% PEG on individual conditioned 30 kDa Nanosep centrifugal device (previously conditioned by wetting with 0.2 mL of distilled water and followed by drying by centrifugation at 12.000 ×g for 10 min) and subjecting the devices to centrifugation at 12.000 ×g for 10 min. The retained protein residues were quantitatively dislodged from the membranes by using 100 *μ*L aliquots of distilled water, and all the washings were pooled together until a 4 mL volume of matrix protein dispersion had been collected. The entire volume of matrix protein dispersion and an equal volume of distilled water were separately mixed with 250 *μ*L of PABA solution and 250 *μ*L of protease solution. A 400 *μ*L volume of each reaction mixture was mixed with 100 *μ*L of APAP-Cys solution, and each type of solution was serially diluted, respectively, with blank stock matrix protein dispersion or distilled water to obtain concentrations of APAP-Cys in the range 1.562–200 *μ*g/mL. Following their incubation at 50°C for 16 hr, the dilutions were filtered through 0.45 *μ*m membrane filters, and a 100 *μ*L aliquot of each filtrate was injected into the liquid chromatograph in duplicate.

The percentage (%) recovery of APAP-Cys from protein matrix was calculated from the peak area ratios gathered in the linearity study. To this effect, the peak area ratios at each dilution for samples prepared in protein matrix were related to those in water. Assuming the peak area ratios for solutions in water to represent 100% recovery, the recovery of APC-Cys from liver matrix protein at each level studied (as a percentage of the peak responses for samples in distilled water) was calculated using the equation (peak area ratio in matrix protein/peak area ratio in water) × 100.

#### 2.5.2. Investigation of the Isolation of APAP-Protein Adducts from Liver Homogenates

Based on the peak area response obtained for an aqueous 10 *μ*g/mL solution of APAP-Cys put through the sample preparation steps used in the linearity test and representing 100% recovery, three sample preparation approaches were examined to determine which one yielded the highest recovery. For this purpose, 100 *μ*L aliquots of a stock solution of APAP-Cys containing 1 mg/mL were added to 400 *μ*L of an incubation mixture obtained by one of three different approaches. The first approach was a centrifugal ultrafiltration method in which 4 mL of matrix protein obtained as described for the method linearity study was treated in the exactly the same manner as for a dilution sample containing a final concentration of APAP-Cys of 10 *μ*g/mL in matrix protein. The second approach was a precipitation method whereby a 4 mL aliquot of drug-free liver homogenate, prepared in phosphate-buffered saline (PBS) pH 7.4 in a ratio of 1 g in 5 mL, was mixed with 400 *μ*L of 10% perchloric acid and centrifuged at 12.000 ×g and 4°C for 20 min to obtain a protein pellet. This pellet was washed with two 200 *μ*L portions of water, resuspended in 4 mL of 3 M urea in PBS pH 8.0, and subjected to sonication in an ultrasonic bath until a uniform dispersion was obtained. This suspension was then processed as described in the linearity study and starting with the consecutive addition of a PABA solution and a protease solution. The third approach was a dialysis method in which a 4 mL aliquot of drug-free liver homogenate, prepared in PBS pH 7.4 using a ratio of 1 g in 5 mL, was placed in a Cellu-Sep H1 tubular dialysis membrane and dialyzed three time against 1000 mL portions of sodium acetate buffer pH 6.5 for periods of 12 hr each. The contents of the dialysis bag were transferred to 4 mL of acetate buffer pH 6.5, and the dispersion was next processed as described in the linearity study and starting with the addition of PABA solution and protease solution.

#### 2.5.3. Intraday and Interday Precision

Samples of APAP-Cys in matrix protein dispersion, prepared as described for the linearity and recovery study and containing 0.156, 0.625, and 1.25 *μ*g of analyte per 100 *μ*L, were injected at three different times in the same day to check for intraday reproducibility or at the same time in three separate days to check for interday reproducibility.

### 2.6. Animal Studies

Groups of 6 male Sprague-Dawley rats, 200–225 g in weight, were maintained on a normal rat chow and filtered tap water for 3 days following their arrival. Following a 12 hr fasting overnight, the animals received a single intraperitoneal (i.p.), 800 mg/kg/2 mL, dose of an APAP solution (100 mg/mL) in 50% PEG 400. Animals treated with an APAP antidote received either N-acetylcysteine (NAC) or taurine (TAU) in physiological saline by the i.p. route, in a 2.4 mmol/kg/2 mL dose, 30 min before APAP. Control animals received only 50% PEG 400 (2 mL) followed 30 min later by water (2 mL). After 6 hr, all the animals were sacrificed by decapitation, and their livers were freeze clamped, surgically excised, and placed in liquid nitrogen until needed.

### 2.7. Liver Sample Preparation

A 1 g portion of frozen liver was mixed with 5 mL of PBS pH 7.4 and homogenized for 30 sec with a hand-held electric tissue homogenizer while kept immersed in an ice bath. The resulting suspension was centrifuged at 12.000 ×g and 4°C for 20 min, and a 500 *μ*L aliquot of the supernatant was placed on a Nanosep centrifugal device with a membrane molecular weight cutoff of 30 kDa (previously conditioned by wetting with 200 *μ*L of distilled water and drying by centrifugation at 12,000 ×g for 10 min) and the device centrifuged at 12.000 ×g for 10 min. The protein retained on the membrane was washed three times with 100 *μ*L portions of distilled water, with centrifugation at 12.000 ×g for 10 min after each washing, and then quantitatively dislodged from the membrane with the aid of a spatula and 100 *μ*L portions of distilled water until a 400 *μ*L volume of protein dispersion had been collected. This protein dispersion was mixed, in succession, with 25 *μ*L of PABA solution (1 mg/mL) and 25 *μ*L of protease solution (8 units/mL), and then incubated at 50°C for 16 hr on a heating block. The digest was transferred to a conditioned Nanosep centrifugal device and centrifuged at 12.000 ×g for 10 min to collect the filtrate. The membrane on the filtration device was rinsed twice with 100 *μ*L portions of distilled water, and the washings and filtrate were pooled together and brought to a final volume of 700 *μ*L with additional distilled water. A 100 *μ*L aliquot of this solution was injected into the liquid chromatograph.

### 2.8. Data Analysis

The experimental results for the animal study are reported as the mean ± SEM for *n* = 6. Statistical comparisons were carried out using Student's *t*-test followed by one-way analysis of variance (ANOVA) and Tukey's test for multiple comparisons. Calculations were performed using a commercially available statistical software program (JMP 8 from SAS Institute Inc., Cary, NC, USA). Differences were considered to be significant when *P* < 0.05.

## 3. Results and Discussion

### 3.1. Isolation of APAP-Protein Conjugates

**Figure 1:**
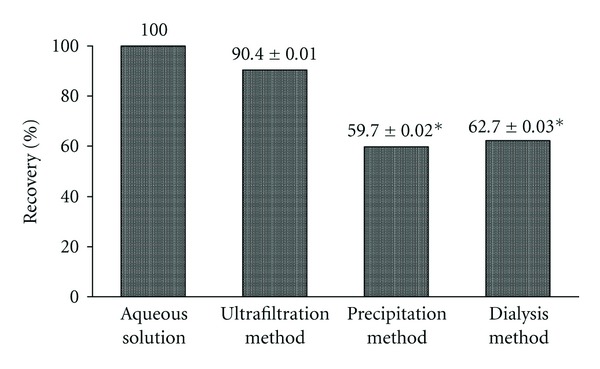
Recovery of APAP-Cys from spiked rat liver matrix protein dispersion by using different methods to isolate the APAP-protein adducts. Values are shown as the mean ± SD for three samples. All values were compared against those obtained for an aqueous solution of APAP-Cys, representing 100% recovery, and where *P < 0.01 versus aqueous solution.

The most appropriate conditions to isolate APAP bound to hepatic proteins via the sulfhydryl side chains of cysteine residues from the liver of animals that had been treated with a supratherapeutic (800 mg/kg) of APAP were investigated using three general approaches: dialysis, chemical precipitation with a deproteinizing solution, and centrifugal ultrafiltration. In each case, the isolated APAP-protein conjugates were subjected to digestion with microbial protease, and the quantity of APAP-Cys thus liberated was measured by the proposed HPLC method. The results originating from these three experiments, carried out in triplicate, are graphically shown in Figure 1.

According to the sample preparation method described by Muldrew et al. [10], a 1 : 10 liver homogenate in 10 mM sodium acetate pH 6.5 is centrifuged at 16.000 ×g and dialyzed against a large volume (4 L) of the homogenizing medium using a dialysis membrane with a molecular mass cutoff of 3500 kDa. Subsequently, the contents of the dialysis bag are precipitated with 40% trichloroacetic acid, rinsed with disodium phosphate pH 7.4, and resuspended in 10 mM sodium acetate pH 6.5. In addition to the need for replacing the dialyzing medium twice (at 9 and 21 hr) and, hence, large volumes of dialyzing medium, this technique requires more than one day for completion. As shown in Figure 1, the recovery of APAP adduct by this method amounted to about 62% of the added amount. Although the exact reason for this deviation from maximum recovery is at present unclear, one potential contributory factor that will certainly require a more detailed investigation is the type of protease used in different laboratories to release APAP-Cys from its anchoring proteins since the activity of different types of protease can vary over a wide pH range [12]. Thus, one would expect a higher yield of APA-Cys using the present type of protease (Pronase E from *Streptomyces griseus*) in a solution buffered with PBS than in one buffered with 10 mM solution of sodium acetate since the pH of the former buffer (i.e., pH 7.4) is closer to the pH declared as optimal (i.e., pH 7.5) by the supplier of this enzyme than is to that of the latter buffer (i.e., pH 6.5).

In an attempt to simplify the isolation of APAP-protein adducts from liver samples, the liver homogenate in PBS pH 7.4 was directly treated with a known protein precipitating agent such as 10% perchloric acid in methanol. The precipitated proteins, separating as a pellet upon centrifugation, were subjected to two cycles of dispersion in water followed by centrifugation and submitted to proteolytic digestion. Although extremely simple and fast, the recovery of APAP-Cys adduct by this protein-separating approach was slightly less (by *∼*3.7%) than that by dialysis (Figure 1), possibly because of the degree of compactness of the protein pellet and of the traces of acid remaining with it, factors which could have, respectively, interfered with the ability of the protease to reach all of the APAP-protein adducts available for proteolysis and for displaying its full hydrolyzing activity.

In the proposed method, APAP-protein adducts were isolated from liver homogenates in PBS pH 7.4 by centrifugal ultrafiltration using a Nanosep centrifugal device. In essence, this device will retain the APAP-protein adduct on the ultrafiltration membrane, which in this case was selected to retain adducts having a molecular weight of 30 kDa and above. The retentive efficiency and, hence, the quantity of APAP-protein adduct left on the membrane, amounted to about 90.0% (Figure 1). In addition to its simplicity and greater efficiency as an isolation technique, processing of a liver homogenate by this technique could be successfully completed in less than 1 hr.

### 3.2. Chromatographic Conditions

**Figure 2:**
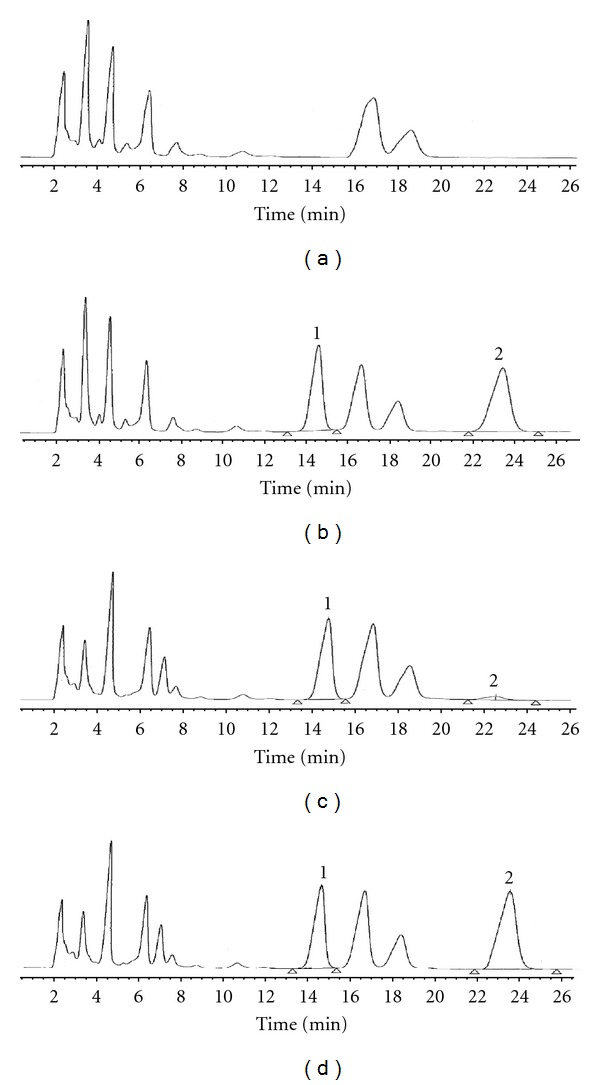
Chromatograms of (a) a liver sample from a rat treated only with physiological saline; (b) a rat liver extract spiked with APAP-Cys (2.5 *μ*g/100 *μ*L) and PABA, the internal standard; (c) a liver extract from a rat treated with APAP (800 mg/kg i.p.); (d) a liver extract from a rat treated with APAP (800 mg/kg i.p.) and spiked *ex vivo* with authentic APAP-Cys (2.5 *μ*g/100 *μ*L of liver extract). Key: 1, PABA, the internal standard; 2, APAP-Cys adduct.

Using photometric detection at 254 nm, PABA, the internal standard, and APAP-CYS, the analyte, injected as part of an aqueous mixture, were observed to elute at about 14.5 and 23.3 min, respectively, (Figure 2(a)). A liver sample from a rat receiving only 50% PEG 400, and put through the ultrafiltration and proteolytic digestion steps, showed chromatographic peaks for endogenous liver components which did not interfere with the peaks of interest (Figure 2(b)). An identical chromatographic pattern was obtained from the analysis of a liver sample from a rat treated with a very high (800 mg/kg) dose of APAP (Figure 2(c)). Confirmatory evidence on the identity of the slower-eluting peak was obtained by spiking the same liver sample with authentic APAP-Cys (*∼*5 *μ*g/100 *μ*L) and observing an enhancement in the size of the peak eluting at *∼*23.0 min (Figure 2(d)).

### 3.3. Linearity, Recovery, and Limits of Detection

The linearity of the proposed method was investigated by preparing two ten-point calibration curves for APAP-Cys, one in blank liver matrix protein and the other in distilled water. For both types of solutions, a linear correlation between peak area responses at 254 nm and on-column concentrations of APAP-Cys in the range 0.078–40.000 *μ*g was observed.

By relating the peak area responses derived from solutions of APAP-Cys in blank liver matrix protein with those from solutions in distilled water, taken as 100% recovery, it was verified that the recovery of APAP-Cys, for on-column concentrations from 0.078 to 40.000 *μ*g, ranged from *∼*83% to *∼*91.0% of the added amount (see Table 1). The linear equations and corresponding coefficient of determination (*r* ^2^) values for solutions of APAP-Cys in liver matrix protein and in distilled were, respectively, *y* = 0.0317 *x* − 0.005  (*r* ^2^ = 0.9996) and *y* = 0.0349 *x* − 0.0037  (*r* ^2^ = 0.998). The lowest concentrations of APAP-Cys that could be detected (i.e., limit of detection) and accurately measured (i.e., limit of quantification) by the proposed method amounted to 0.06 *μ*g and 0.14 *μ*g (on column), respectively, at a signal-to-noise ratio of at least 2 : 1.

### 3.4. Precision and Accuracy

Intraday variability was assessed by using the proposed method to analyze samples of APAP-Cys, added as a spike to distilled water and to liver matrix protein, at three different levels (i.e., 0.156, 0.625, 1.250 *μ*g) and at three different times within the same day. As shown in Table 2, the RSD values ranged from 0.52 to 0.91% for samples in water and from 0.63% to 1.02% for samples in matrix protein. Evaluation of method interday variability was carried out using the sample solutions used in the intraday variability study. In this case, each sample solution was analyzed once daily, for three consecutive days. As shown in Table 3, the RSD values for samples in water and in matrix protein were in the range 0.60 to 0.87% and 1.05 to 1.32%, respectively.

### 3.5. Tissue Sample Analysis to Evaluate the Effect of APAP Antidotes on APAP-Cys Formation

To test the suitability of the proposed HPLC method for measuring the levels of APAP-Cys adduct formation in the liver in the course of evaluating pharmacological agents with potential for use as antidotes against APAP overdose, rats were intraperitoneally treated with a dose of APAP (800 mg/kg) reported to be hepatotoxic in this animal species [13]. Two additional groups of rats received NAC or TAU, in a 2.4 mmol/kg i.p. dose, 30 min before a treatment with APAP. While NAC is the most widely accepted antidote for cases of APAP overdose [14, 15], TAU has shown prophylactic and therapeutic effects in APAP-induced hepatic injury, apoptosis, and necrosis [13]. In the absence of an antidote, a discernible peak with a retention time (*∼*22.5 min) comparable with that of an authentic sample of APAP-Cys, and yielding an area equivalent to 19 *μ*g/g of liver sample, was observed at 6 hr after a treatment with APAP. In contrast, this chromatographic peak was not detected in livers from rats treated with either NAC or TAU ahead of APAP.

## 4. Conclusions

The HPLC method presented here should prove useful in animal studies aimed at verifying the formation of APAP-protein adducts following a treatment with a toxic dose of APAP as well as the effect of compounds and substances with potential for use as an antidote against APAP overdosing. Salient features of this method are a sample preparation approach based on centrifugal ultrafiltration that minimizes procedural steps and reagents and permits the isolation of APAP-protein adducts in a rapid manner and with a high degree of purity and yield. Except for an overnight protease digestion step, one complete analysis can be completed in less than 2 hr. Hence, the proposed method will be quite suited in APAP-related studies with small animals and requiring the analysis of multiple samples.

## Figures and Tables

**Table 1:** Results of a recovery study of APAP-Cys from spiked a rat liver matrix protein dispersion^a,b^.

Amount added (*μ*g)	Amount found (*μ*g)	Mean recovery (%)
0.078	0.065 ± 0.003	83.3
0.156	0.136 ± 0.001	87.1
0.312	0.300 ± 0.002	88.5
0.625	0.547 ± 0.002	87.5
1.250	1.108 ± 0.002	88.6
2.500	2.262 ± 0.001	90.5
5.000	4.488 ± 0.002	89.8
10.000	8.906 ± 0.001	89.1
20.000	18.129 ± 0.001	90.6
40.000	36.380 ± 0.001	91.0

^a^Values for amount found represent the mean ± SD for *n* = 3 samples.

^b^Mean recoveries were calculated by comparing the peak area ratio responses of spiked rat liver samples with those of equipotent samples in distilled water and regarded as 100% recovery.

**Table 2:** Results of the intraday variability study on the proposed HPLC method based on three different concentrations of APAP-Cys added to distilled water and to blank rat liver matrix protein dispersion^a,b^.

APAP-Cys injected (*μ*g)	Peak area ratio (mean ± SD, *n* = 3)	RSD (%)
Samples in distilled water		

0.156	0.005 ± 0.002	0.91
0.625	0.021 ± 0.001	0.72
1.250	0.042 ± 0.001	0.52

Samples in rat liver matrix protein dispersion		

0.156	0.004 ± 0.001	1.02
0.625	0.018 ± 0.001	0.68
1.25	00.040 ± 0.003	0.63

^a^Each sample was analyzed at 4 hr intervals three times on the same day.

^b^RSD is the relative standard deviation.

**Table 3:** Results of the intraday variability study on the proposed HPLC method based on three different concentrations of APAP-Cys added to distilled water and to blank rat liver matrix protein dispersion^a^.

APAP-Cys injected (*μ*g)	Peak area ratio (mean ± SD, *n* = 3)	RSD (%)
Samples in distilled water		

0.156	0.005 ± 0.002	0.87
0.625	0.020 ± 0.001	0.60
1.250	0.042 ± 0.001	0.61

Samples in rat liver matrix protein dispersion		

0.156	0.004 ± 0.001	1.15
0.625	0.017 ± 0.001	1.32
1.250	0.039 ± 0.003	1.05

^a^Each sample was analyzed once a day for three consecutive days.
